# The Effects of Low-Level Laser Therapy on Wound Healing and Pain Management in Skin Wounds: A Systematic Review and Meta-Analysis

**DOI:** 10.7759/cureus.72542

**Published:** 2024-10-28

**Authors:** Nadia Taha, Hasan Daoud, Tahira Malik, Jeevith Shettysowkoor, Shafiq Rahman

**Affiliations:** 1 Department of Plastic Surgery, Leeds Teaching Hospitals National Health Service (NHS) Trust, Leeds, GBR; 2 Department of Orthopaedics, Hull Royal Infirmary, Hull, GBR; 3 Department of Plastic Surgery, Leeds General Infirmary, Leeds, GBR; 4 Department of Orthopaedic Surgery, Harrogate District Hospital, Harrogate, GBR; 5 Department of Plastic Surgery, Northern General Hospital, Sheffield Teaching Hospitals National Health Service (NHS) Foundation Trust, Sheffield, GBR

**Keywords:** dfu, diabetic foot ulcers, lllt, low-level laser therapy, wound healing

## Abstract

Low-level laser therapy (LLLT) is a non-invasive application of non-thermogenic light that is proven to promote tissue healing and alleviate pain. The authors aim to conduct the first meta-analysis, evaluating the effects of LLLT on wound healing and pain in skin wounds by comparing it to skin wounds not treated with LLLT. The Preferred Reporting Items for Systematic Reviews and Meta-Analyses guidelines were followed by searching the electronic databases. Eighteen randomised controlled trials that met the inclusion criteria were included in the study. Six hundred seventy skin wounds were analysed in the study. The primary outcome measures were the percentage reduction in wound size and the rate of complete wound healing. The secondary outcome measures included the visual analogue scale (VAS) for pain and the perineal pain score in episiotomy wounds. The percentage reduction of wound size in the LLLT group was significantly greater than that in the control group (95% confidence interval, CI, 13.93-37.70; p < 0.0001). In addition, the rate of wound healing was significantly greater in the LLLT group (95% CI, 2.32-16.70; p = 0.0003). LLLT has been shown to reduce pain, with the VAS scores for pain being significantly lower in the LLLT group after treatment (95% CI, -2.52 to -0.19; p = 0.02). The authors present the first meta-analysis within the literature showing the effects of LLLT on wound healing and pain in skin wounds. Higher quality trials are recommended to enhance the current evidence base.

## Introduction and background

Low-level laser therapy (LLLT) was introduced in 1966 by Endre Mester, a professor of surgery in Budapest [1]. It is a non-invasive application of non-thermogenic light to stimulate biological activity. The light is absorbed, causing chemical changes within the body, which can result in therapeutic outcomes on the biological system to promote tissue healing and regeneration, alleviate pain, and reduce inflammation. The wavelength used is in the range of 300-10,600 nm, with the delivery of 1-4 J/cm^2^ and an output power between 10 and 90 mW, and is therefore not comparable to other forms of laser therapy such as cutting, thermal tissue coagulation and ablation [2,3]. Absorption of the light increases adenosine triphosphate production and the induction of transcription factors through action on the mitochondria. This leads to increased cell proliferation and migration of cells, including fibroblasts, which play a crucial role in wound healing. LLLT also promotes an increase in collagen synthesis and the formation of granulation tissue, thereby encouraging tissue healing and regeneration, alleviating pain and reducing inflammation [2]. The irradiation is used in different wavelengths and energy densities to accelerate wound healing. LLLT is also used in non-skin wounds, including musculoskeletal pain and dermatological and dental conditions [4]. LLLT can be referred to as photobiomodulation therapy and includes non-ionising forms of light therapy, including lasers, light-emitting diodes (LED), and broadband light in the infrared spectrum [5].

This meta-analysis is the first in the literature to examine the effects of LLLT on wound healing and pain relief in human skin wounds. LLLT has been utilised in a variety of skin wounds, and this analysis will cover its effects on wounds from leprosy ulcers, bariatric surgery, hernia repairs, thyroidectomy scars, burns, full-thickness skin graft (FTSG) donor sites, episiotomies and diabetic foot ulcers (DFUs). Numerous studies have highlighted the therapeutic benefits of LLLT in accelerating the healing of DFUs, which are a common complication among diabetic patients. The lifetime risk of developing DFUs in diabetics can be as high as 25%, with a five-year mortality rate of up to 40% [3,6]. DFUs are often poorly managed with conventional treatments, significantly impacting patients' quality of life and posing risks of infection and amputation [7].

A secondary goal of this study is to elucidate information that may be useful to clinicians in developing treatment guidelines. There is an abundance of studies and analyses looking into LLLT on wound healing in non-human subjects. Studies have demonstrated that the effectiveness of LLLT in wound healing is dependent on treatment parameters such as wavelength, output power and energy density [8]. In general, output powers between 5 and 50 mW at 1-4 Jcm^2^ have been most effective at stimulating tissue repair [1]. Conventional management of skin wounds includes debridement, antibiotics and saline irrigation. This study aims to assess if LLLT is effective as an adjunct to traditional treatment pathways for wounds. The review has not been registered. The review authors have no competing interests.

## Review

Methods

This systematic review and meta-analysis was designed using the Preferred Reporting Items for Systematic Reviews and Meta-Analyses (PRISMA) statement standards [9]. This study presents no ethical concerns, and there are no reported conflicts of interest. Furthermore, it is important to note that no external funding was received for this research.

Study Types

The eligibility criteria for this study include all randomised controlled trials (RCTs) on patients who received LLLT on skin wounds where wound healing and pain response were assessed. Male and female patients of all ages were included. There were no exclusion criteria on co-morbid status, the type of skin wound or the type of LLLT used. Case series, case reports, letters, non-randomised control studies and studies not reported in English were excluded from this review.

Primary and Secondary Outcomes

The primary outcome measures were the percentage reduction in wound size and the rate of complete wound healing. The secondary outcome measure included the visual analogue scale (VAS) for pain and the perineal pain score in episiotomy wounds.

Literature Search Strategy

Two authors, N.T. and H.D., independently searched the electronic databases Medical Literature Analysis and Retrieval System Online, PubMed, Excerpta Medica dataBASE, Google Scholar, Cumulative Index to Nursing and Allied Health Literature and the Cochrane Central Register of Controlled Trials. The World Health Organization International Clinical Trials Registry (http://apps.who.int/trialsearch/), ClinicalTrials.gov (http://clinical-trials.gov/) and the International Standard Randomised Controlled Trial Number registry (http://www.isrctn.com/) were also searched. The terms used in the search tool included the following: 'low level laser therapy, LLLT, photobiomodulation, laser emitting diode, LED, wound healing and randomised control trial'. These search terms were paired with the adjuncts 'or' and 'and'. The reference lists of relevant articles were screened for optimal selection.

Study Selection

Two authors, N.T. and H.D., independently reviewed the abstracts of the articles generated in the literature search. The relevant articles were extracted, and the full text was assessed. A third author, J.S., reviewed the articles for any discrepancies in the selection.

Data Extraction and Management

Relevant data from the papers were extracted by the authors from the studies and collated in a spreadsheet. Initially, this was tested with random articles and adjusted to include the relevant data. The spreadsheet comprised the first author, year of publication, total number of wounds per group, percentage change in the wound size before and after treatment, the complete healing rate of the wound, VAS score, pressure ulcer scale for healing and the wound healing index.

Statistical Analysis

Extracted data were entered into RevMan version 8.4.1., Cochrane Collaboration, London, UK, by the authors individually using the random effects model. The results were displayed in forest plots with 95% confidence intervals (CIs), and the mean difference was used to analyse the continuous data. The heterogeneity of the studies was assessed using Cochran's Q test (χ^2^), and any discrepancies were quantified with the calculation of the I2 assessment score. The I2 score was interpreted as follows: 0%-25%, low heterogeneity; 25%-75%, moderate heterogeneity; and 75%-100%, high heterogeneity.

Results

Literature Search Results

**Figure 1:**
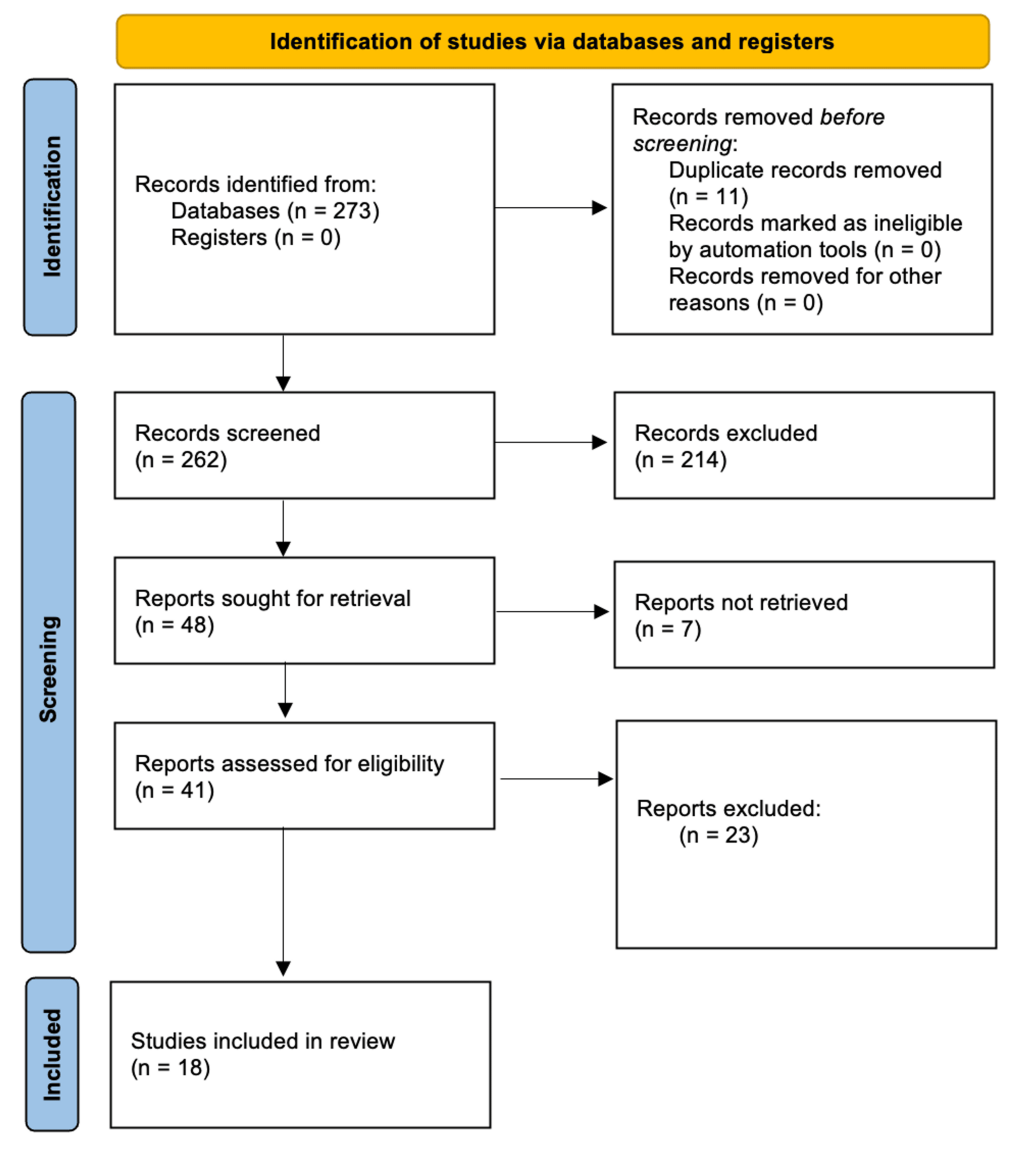
PRISMA flow diagram PRISMA: Preferred Reporting Items for Systematic Reviews and Meta-Analyses Credit: The image has been created by the authors

The two authors independently conducted a literature search, identifying 18 articles. From this selection, 18 studies were selected that met the eligibility criteria. The PRISMA flow diagram in Figure 1 demonstrates the literature search process.

Description of Studies

Table 1 amalgamates the baseline characteristics of the studies. The data analysis in this study includes 670 skin wounds, of which 216 are diabetic foot ulcers (DFUs), 50 leprosy ulcers, 18 burn wounds, 18 FTSG donor site wounds, 182 episiotomy wounds, 30 sternotomy wounds, 85 bariatric surgery wounds, 28 hernia repair skin wounds and 43 thyroidectomy scar wounds.

**Table 1:** Amalgamation table GaAlAs: gallium-aluminium-arsenate laser

Study	Number of participants (laser/control)	Type of wound	Type of laser (wavelength)	Output power	Energy density	Application site	Frequency of application	Laser emitting frequency	Control group treatment	Laser group additional treatment
Haze et al. [10]	10/10	Diabetic foot ulcer	Ga-Al-As laser Near-infrared (808 nm)	250 mW	8.8 Jcm^-2^	Diabetic foot ulcer	At home daily until complete ulcer closure for 12 weeks	Pulsed 8 minutes per area	Do	Do
Kim et al. [11]	21/22	Thyroidectomy scar	Diode laser (830 nm)	-	4.5 Jcm^2^	Neck area	Daily for 30 minutes	10 cycles continuous from 80 to 180 seconds	Sham laser	-
Helmy et al. [12]	15/15	Longitudinal median sternotomy wound	Probe laser device Petra, Laserklasse 2 M, Germany (660 nm)	-	6 Jcm^2^	Five to eight points on the sternotomy scar 2 cm apart	One session 3 times a week for 4 weeks	Each spot 60 seconds for 5-10 minutes	Cardiac rehabilitation programme and sternal precautions	Cardiac rehabilitation programme and sternal precautions
Kazemikhoo et al. [13]	9/9	Grade 3 burn ulcer covered by a split-thickness skin graft	Red diode laser (655 nm)	150 mW	2 Jcm^-2^	Bed of the ulcer and the margins	Once a day for 7 days	Continuous	Do	Do
De Alencar Fonseca Santos et al. [4]	9/9	Diabetic foot ulcer	Red diode laser (660 nm)	30 mW	6 Jcm^2^	Edge of the lesion	Once every 48 hours	Continuous	Physiological solution 9%, hydrogel 2 mg on the wound bed with wool and gauze, every 48 hours	Physiological solution 9%, hydrogel 2 mg on the wound bed with wool and gauze, every 48 hours
Vaghardoost et al. [14]	9/9	Split-thickness skin graft donor site	Red diode laser (655 nm)	150 mW	2 Jcm^2^	Donor site	Immediately after surgery, days 3, 5 and 7	Continuous	Do	Do
Mathur et al. [15]	15/15	Diabetic foot ulcer	Diode laser (660 nm)	50 mW	3 Jcm^2^	Five to eight spatially separated points (ulcer floor and edge)	Daily for 15 days	Continuous for 60 seconds	Daily wet saline or betadine dressings, antibiotic treatment, contact cast immobilisation and slough excision when required	Daily wet saline or betadine dressings, antibiotic treatment, contact cast immobilisation and slough excision when required/after laser, the wound was covered with a moist dressing
Alvarenga et al. [16]	29/25	Episiotomy	GaAIAs laser (780 nm)	20 mW	5 Jcm^2^	9 points on the episiotomy equally distributed on the left and right side of the suture line 1 cm away from the edge of the wound	Three sessions of laser therapy: 6-10 hours postpartum, 20-24 hours postpartum and 40-48 hours postpartum	90 seconds per session	-	-
Ojea et al. [17]	43/42	Bariatric surgery wound	Diode laser (808 nm)	100 mW	10 Jcm^2^	Abdominal wound 1 cm apart	Immediately post-operative and on days 1 and 7	Continuous 20 seconds per point	-	-
Sandoval Ortíz et al. [18]	9/9	Diabetic foot ulcer	Semiconductor laser (685 nm)	30 mW	1.5 Jcm^2^	Wound edges and bed 1 cm apart	Treated 3 times a week for 16 weeks or until wound closure	Continuous	Daily saline wash and sharp debridement of necrotic tissue as required, maintaining a moist environment	-
Santos et al. [19]	38/38	Episiotomy wound	Red diode laser (660 nm) and infrared laser (780 nm)	35 mW	8.8 Jcm^2^	3 points on the wound	Single session 6-56 hours post-partum	-	Laser procedure with the emission of irradiation	-
Santos et al. [20]	26/26	Episiotomy wound	Red diode laser (660 nm)	15 mW	3.8 Jcm^2^	3 points on the wound	Treated after sutures were placed, up to 2 hours post-partum, between 20 and 24 hours post-partum and between 40-48 hours post-partum	-	Three treatment sessions without the emission of radiation	-
Kajagar et al. [21]	34/34	Diabetic foot ulcer	Multidiode cluster probe	60 mW	2-4 Jcm^-2^	Ulcer floor and edge	Daily for 15 days	-	Daily wet saline or betadine dressings, antibiotic treatment, contact cast immobilisation and slough excision when required	Daily wet saline or betadine dressings, antibiotic treatment, contact cast immobilisation and slough excision when required
Landau et al. [22]	10/6	Diabetic foot ulcer	Broadband light (400-800 nm)	180 mW	-	Entire wound	Twice a day for 4 minutes at a distance of 2 cm	-	Non-healing light fluency 10 mW/cm^2^, daily cleaning and debridement as required, application of a pad of gauze soaked with saline and wound dressings	Daily cleaning and debridement as required, application of a pad of gauze soaked with saline and wound dressings
Kaviani et al. [23]	13/10	Diabetic foot ulcer	BTL (685 nm)	50 mW	10 Jcm^-2^	Ulcer surface	Six times a week for 2 weeks, then every other day up to complete healing	200 seconds of illumination	Antibiotic treatment and slough excision when required	Sham irradiation, and antibiotic treatment and slough excision when required
Barreto and Salgado [24]	25/25	Ulcers in leprosy patients	Indium-gallium-aluminium-phosphide semiconductor visible red light (660 nm)	40 mW	2-4 Jcm^-2^	Wound bed and edges	Three times a week for 12 weeks	Continuous	Simple saline dressings with sterile gauze and 1% silver sulfadiazine cream	Simple saline dressings with sterile gauze and 1% silver sulfadiazine cream
Carvalho et al. [25]	14/14	Inguinal hernia scar	GaAIAs diode laser (830 nm)	40 mW	1.04-13 Jcm^-2^	10 points across the length of the scar	Treatment on the day of surgery and then on days 3, 5 and 7	26 seconds per point	-	-
Minatel et al. [26]	13/10	Diabetic foot ulcer	Dynatron Solaris 7051 phototherapy, Salt Lake City, UT (660-890 nm)	100 mW	3 Jcm^-2^	Ulcer surface	Two times a week for 90 days or until complete healing of the ulcer	Each spot size was treated for 30 seconds	Saline wash and ulcers cleaned with 1% sulfadiazine cream and treated with placebo phototherapy of <1.0 Jcm^-2 ^twice a week	Saline wash and ulcers cleaned with 1% sulfadiazine cream after laser sessions

Risk of Bias Assessment

The Cochrane Collaboration tool (Cochrane Collaboration, London, UK), outlined in Table 2, was used to assess the risk of bias for the RCTs by the two authors independent of each other.

**Table 2:** Cochrane Collaboration tool

Study	Bias	Authors judgement	Support for judgement
Haze et al. [10]	Random sequence generation (selection bias)	Low risk	Computer-generated randomisation list
	Allocation concealment (selection bias)	Low risk	A researcher who was not an assessor allocated groups to participants
	Blinding of participants and personnel (performance bias)	Low risk	Patient caregivers and personnel were blinded. A sham laser was used
	Blinding of outcome assessment (detection bias)	Low risk	Evaluators were blinded
	Incomplete outcome data (attrition bias)	Low risk	All outcome data reported
	Selective reporting (reporting bias)	Low risk	Study protocol available with no missing outcomes
Kim et al. [11]	Random sequence generation (selection bias)	Low risk	Participants allocated numbers with a random number generator
	Allocation concealment (selection bias)	Low risk	A researcher who was not an assessor allocated groups to participants
	Blinding of participants and personnel (performance bias)	Low risk	Both groups received an identical laser therapy device
	Blinding of outcome assessment (detection bias)	Low risk	The assessors were blinded
	Incomplete outcome data (attrition bias)	Low risk	All outcome data reported
	Selective reporting (reporting bias)	Low risk	Study protocol available with no missing outcomes
Helmy et al. [12]	Random sequence generation (selection bias)	Unclear risk	Randomisation method not specified
	Allocation concealment (selection bias)	Unclear risk	The allocation concealment method is not specified
	Blinding of participants and personnel (performance bias)	High risk	Participants were not blinded to treatment, and blinding of personnel was not specified
	Blinding of outcome assessment (detection bias)	Unclear risk	Blinding of outcome assessment not specified
	Incomplete outcome data (attrition bias)	Low risk	All outcome data reported
	Selective reporting (reporting bias)	Low risk	Study protocol available with no missing outcomes
Kazemikhoo et al. [13]	Random sequence generation (selection bias)	Low risk	All patients received both the control and intervention
	Allocation concealment (selection bias)	Low risk	All patients received both the control and intervention
	Blinding of participants and personnel (performance bias)	Low risk	All patients received both the control and intervention
	Blinding of outcome assessment (detection bias)	Low risk	All patients received both the control and intervention
	Incomplete outcome data (attrition bias)	Low risk	All outcome data reported
	Selective reporting (reporting bias)	Low risk	Study protocol available with no missing outcomes
De Alencar Fonseca Santos et al. [4]	Random sequence generation (selection bias)	Unclear risk	Randomisation method not specified
	Allocation concealment (selection bias)	Unclear risk	The allocation concealment method is not specified
	Blinding of participants and personnel (performance bias)	High risk	Blinding of personnel not specified. Participants were not blinded
	Blinding of outcome assessment (detection bias)	Unclear risk	Blinding of outcome assessment not specified
	Incomplete outcome data (attrition bias)	Low risk	All outcome data reported
	Selective reporting (reporting bias)	Low risk	Study protocol available with no missing outcomes
Vaghardoost et al. [14]	Random sequence generation (selection bias)	Unclear risk	Randomisation method not specified
	Allocation concealment (selection bias)	Unclear risk	The allocation concealment method is not specified
	Blinding of participants and personnel (performance bias)	Low risk	Participants and personnel blinded
	Blinding of outcome assessment (detection bias)	Unclear risk	Blinding of outcome assessment not specified
	Incomplete outcome data (attrition bias)	Low risk	All outcome data reported
	Selective reporting (reporting bias)	Low risk	Study protocol available with no missing outcomes
Mathur et al. [15]	Random sequence generation (selection bias)	Unclear risk	Randomisation technique not specified
	Allocation concealment (selection bias)	Unclear risk	The allocation concealment method is not specified
	Blinding of participants and personnel (performance bias)	High risk	Unable to blind personnel and participants
	Blinding of outcome assessment (detection bias)	High risk	Not blinded
	Incomplete outcome data (attrition bias)	Low risk	All outcome data reported
	Selective reporting (reporting bias)	Low risk	Study protocol available with no missing outcomes
Alvarenga et al. [16]	Random sequence generation (selection bias)	Low risk	A statistician prepared envelopes with participants' group allocation
	Allocation concealment (selection bias)	Low risk	The envelopes were handed to the main researcher immediately before the laser therapy
	Blinding of participants and personnel (performance bias)	Low risk	The control group received a laser tip without the laser being emitted, and the assessor was blinded to which laser was administered
	Blinding of outcome assessment (detection bias)	Low risk	The assessor was blinded to the treatment given
	Incomplete outcome data (attrition bias)	Low risk	All outcome data reported
	Selective reporting (reporting bias)	Low risk	Study protocol available with no missing outcomes
Ojea et al. [17]	Random sequence generation (selection bias)	Low risk	An individual who was blinded to the patients tossed a coin to decide on group allocation
	Allocation concealment (selection bias)	Unclear risk	The allocation concealment method is not specified
	Blinding of participants and personnel (performance bias)	High risk	Unable to blind participants
	Blinding of outcome assessment (detection bias)	Low risk	The assessor was blinded to the treatment given
	Incomplete outcome data (attrition bias)	Low risk	All outcome data reported
	Selective reporting (reporting bias)	Low risk	Study protocol available with no missing outcomes
Sandoval Ortíz et al. [18]	Random sequence generation (selection bias)	Unclear risk	Randomisation technique not specified
	Allocation concealment (selection bias)	Unclear risk	The allocation concealment method is not specified
	Blinding of participants and personnel (performance bias)	High risk	Unable to blind personnel and participants
	Blinding of outcome assessment (detection bias)	High risk	Not blinded
	Incomplete outcome data (attrition bias)	Low risk	All outcome data reported
	Selective reporting (reporting bias)	Low risk	Study protocol available with no missing outcomes
Santos et al. [19]	Random sequence generation (selection bias)	Low risk	Randomisation of the subjects was done using a randomisation table that identified each woman by a numerical code
	Allocation concealment (selection bias)	Low risk	Each number in the list was placed in an opaque sealed envelope and numbered, and the principal investigator opened them
	Blinding of participants and personnel (performance bias)	Low risk	Participants and personnel were blinded as all received laser therapy
	Blinding of outcome assessment (detection bias)	Unclear risk	Method not specified
	Incomplete outcome data (attrition bias)	Low risk	All outcome data reported
	Selective reporting (reporting bias)	Low risk	Study protocol available with no missing outcomes
Santos et al. [20]	Random sequence generation (selection bias)	Unclear risk	Randomisation method not specified
	Allocation concealment (selection bias)	Unclear risk	The allocation concealment method is not specified
	Blinding of participants and personnel (performance bias)	Low risk	Participants were blinded as both groups received laser therapy
	Blinding of outcome assessment (detection bias)	High risk	The assessor was not blinded to the study group
	Incomplete outcome data (attrition bias)	Low risk	All outcome data reported
	Selective reporting (reporting bias)	Low risk	Study protocol available with no missing outcomes
Landau et al. [22]	Random sequence generation (selection bias)	Low risk	Randomisation performed by a person who was not involved in the evaluation of the study
	Allocation concealment (selection bias)	Low risk	Participants were all given identical laser devices
	Blinding of participants and personnel (performance bias)	Low risk	Participants received a non-treatment dose of laser. Personnel not blinded
	Blinding of outcome assessment (detection bias)	Low risk	Surgical team blinded
	Incomplete outcome data (attrition bias)	Low risk	All outcome data reported
	Selective reporting (reporting bias)	Low risk	Study protocol available with no missing outcomes
Kajagar et al. [21]	Random sequence generation (selection bias)	Low risk	Participants were randomised with a computer-generated number
	Allocation concealment (selection bias)	High risk	Participants were informed about the group allocated to
	Blinding of participants and personnel (performance bias)	High risk	Participants and personnel not blinded to the group allocated
	Blinding of outcome assessment (detection bias)	High risk	Not blinded
	Incomplete outcome data (attrition bias)	Low risk	All outcome data reported
	Selective reporting (reporting bias)	Low risk	Study protocol available with no missing outcomes
Kaviani et al. [23]	Random sequence generation (selection bias)	Low risk	Randomisation list prepared by an independent statistician by using a computer randomisation generator
	Allocation concealment (selection bias)	Low risk	Participants were all given identical laser devices
	Blinding of participants and personnel (performance bias)	Low risk	Participants in the control group received a sham laser
	Blinding of outcome assessment (detection bias)	Unclear risk	Not specified
	Incomplete outcome data (attrition bias)	Low risk	All outcome data reported
	Selective reporting (reporting bias)	Low risk	Study protocol available with no missing outcomes
Barreto and Salgado [24]	Random sequence generation (selection bias)	Low risk	Randomisation with computer-generated software
	Allocation concealment (selection bias)	Low risk	The allocation concealment method is not specified
	Blinding of participants and personnel (performance bias)	High risk	Unable to blind participants and personnel due to the red colour of the laser
	Blinding of outcome assessment (detection bias)	High risk	Unable to blind the assessors but used one researcher to assess all ulcers
	Incomplete outcome data (attrition bias)	Low risk	All outcome data reported
	Selective reporting (reporting bias)	Low risk	Study protocol available with no missing outcomes
Carvalho et al. [25]	Random sequence generation (selection bias)	Unclear risk	Randomisation method not specified
	Allocation concealment (selection bias)	Low risk	Participants were given a stamped envelope with either number 1 or 2
	Blinding of participants and personnel (performance bias)	High risk	Unable to blind participants and personnel due to the use of a red laser or no laser
	Blinding of outcome assessment (detection bias)	High risk	Unable to blind assessors
	Incomplete outcome data (attrition bias)	Low risk	All outcome data reported
	Selective reporting (reporting bias)	Low risk	Study protocol available with no missing outcomes
Minatel et al. [26]	Random sequence generation (selection bias)	Low risk	The study reports complete randomisation but does not specify the method
	Allocation concealment (selection bias)	Low risk	All participants received treatment from an identical laser. The control group received a non-treatment dose
	Blinding of participants and personnel (performance bias)	Low risk	Participants and personnel were blinded until the completion of the study
	Blinding of outcome assessment (detection bias)	Low risk	An identical light probe was used in both treatment and control groups
	Incomplete outcome data (attrition bias)	Low risk	All outcome data reported
	Selective reporting (reporting bias)	Low risk	Study protocol available with no missing outcomes

Primary Outcome

**Figure 2:**
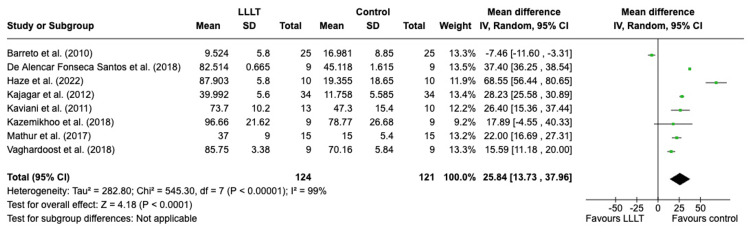
A forest plot demonstrating the percentage reduction in the wound size of all skin wounds LLLT: low-level laser therapy; SD: standard deviation; IV: intravenous; CI: confidence interval Credit: The forest plot has been created by the authors using RevMan version 8.4.1

*Percentage reduction in wound size of all wounds*:* *Figure 2 shows the eight studies measuring the percentage reduction in wound size from before and after treatment [4,10,13,14,21,23,24]. This included 263 of the 670 analysed wounds, with 133 receiving LLLT and 130 in the control group. The wounds analysed were ulcers, burns and skin graft donor sites. The percentage reduction in the LLLT group was significantly greater than that in the control group (95% CI, 13.93-37.70; p < 0.0001). The results were statistically significant when using the random effects model. However, there is a high heterogeneity in the results, with the I2 score at 99%, meaning there is a lot of variation within the results.

**Figure 3:**
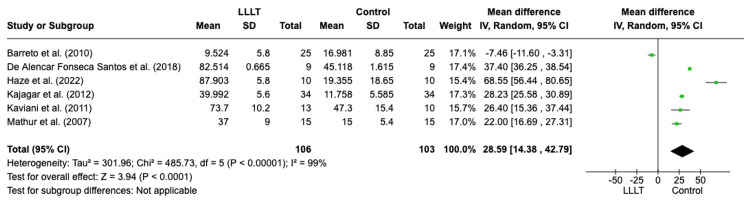
A forest plot demonstrating the percentage reduction in the wound size of all ulcers LLLT: low-level laser therapy; SD: standard deviation; IV: intravenous; CI: confidence interval Credit: The forest plot has been created by the authors using RevMan version 8.4.1

*Percentage reduction in wound size of all ulcers*:* *Six studies looked at the percentage reduction in size of all ulcers, as shown in Figure 3 [4,10,15,21,23,24]. The ulcers were DFUs and leprosy-induced ulcers. The results for percentage reduction in the size of the ulcers are statistically significant, with a 95% CI of 14.38-42.79 and p < 0.0001. The six studies included 209 wounds, with 106 in the laser group and 103 in the control. Similar to the results of percentage reduction in all wounds in Figure 3, there is a high heterogeneity with the I2 score again at 99%.

**Figure 4:**
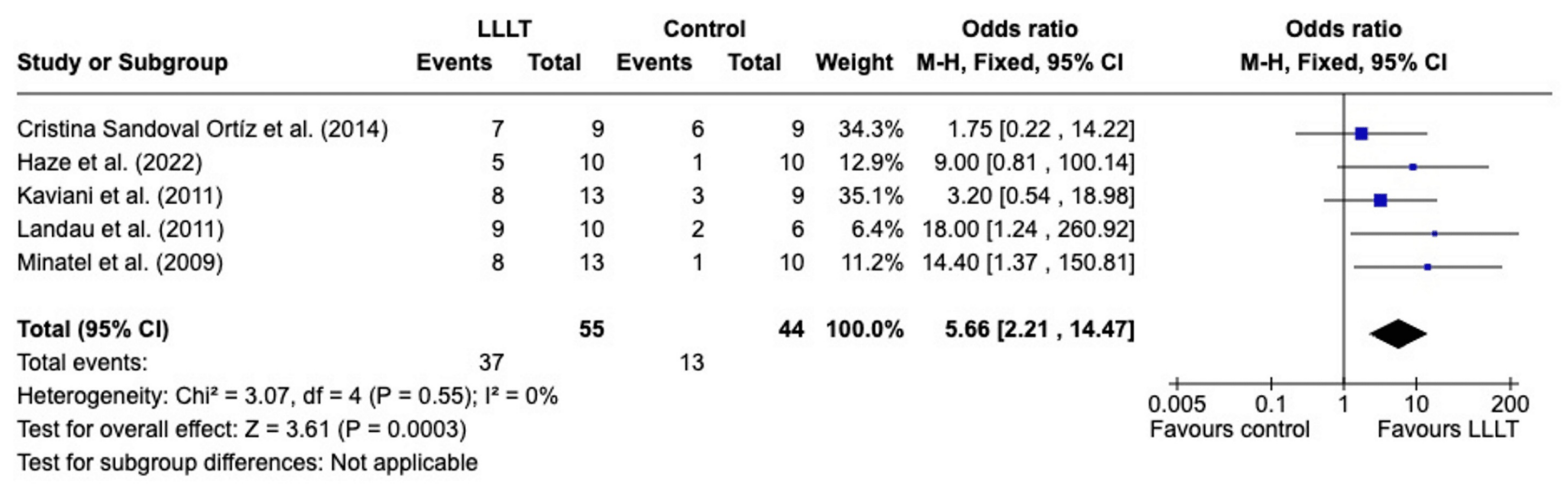
A forest plot to show the complete wound healing rate LLLT: low-level laser therapy; M-H: Mantel-Haenszel; CI: confidence interval Credit: The forest plot has been created by the authors using RevMan version 8.4.1

*Complete wound healing*: Complete wound healing rates are displayed in Figure 4 (a forest plot to show the complete wound healing rate), including five studies assessing DFUs [10,18,22,23,26]. In the work by Haze et al., complete wound healing is defined as wound closure equal to or over 90% and, in other studies, as complete closure of the wound [10]. The rate of wound healing was greater in the treatment group than that in the control group, with statistically significant results (p = 0.0003; 95% CI, 2.32-16.70) using the random effects model. This included 99 wounds, with 55 in the treatment and 44 in the control. There was no heterogeneity between the studies (I2 = 0%).

Secondary Outcome

**Figure 5:**
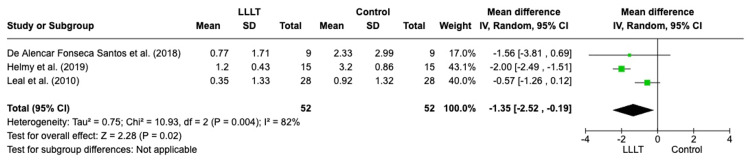
A forest plot demonstrating the VAS scores for pain LLLT: low-level laser therapy; SD: standard deviation; IV: intravenous; CI: confidence interval Credit: The forest plot has been created by the authors using RevMan version 8.4.1

*Visual analogue scale*: The VAS scores for pain are displayed in Figure 5 (a forest plot demonstrating the VAS scores for pain), which are reported in three studies [4,12,25]. The VAS is a subjective scale from 0 to 10 on a 10-cm line, where 0 represents 'no pain' and 10 is 'worst pain' [27]. The wounds involved include DFUs, sternotomy wounds and inguinal hernia scar wounds. The results demonstrate lower VAS scores for pain in the LLLT group after treatment and a statistically significant difference (95% CI, -2.52 to -0.19; p = 0.02).

**Figure 6:**
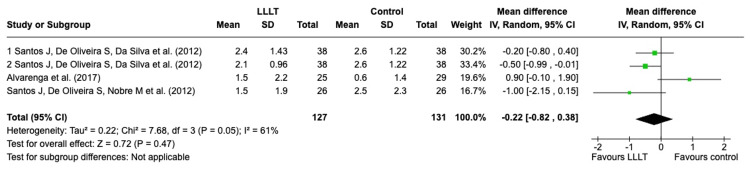
A forest plot displaying the results for the perineal pain scores in the studies with episiotomy wounds LLLT: low-level laser therapy; SD: standard deviation; IV: intravenous; CI: confidence interval Credit: The forest plot has been created by the authors using RevMan version 8.4.1

*Perineal pain score*: Three studies reported the perineal pain scores, which are reported in Figure 6 (a forest plot displaying the results for the perineal pain scores in the studies with episiotomy wounds) [16,19,20]. The results suggest that LLLT is not beneficial in alleviating pain in episiotomy wounds, with statistically insignificant results (p = 0.47).

Discussion

Unlike other forms of medical laser therapy, LLLT is non-invasive and does not have thermal or ablative mechanisms, making it a safe and low-risk treatment option [2]. This is the first meta-analysis within the literature assessing the efficacy of LLLT on wound healing and pain response in a wide variety of skin wounds. This analysis demonstrates that LLLT results in a significant reduction in wound size (p < 0.0001), resulting in an increased rate of complete wound healing (p = 0.0003) and pain reduction (p = 0.02). The type of low-level laser used in the studies varied. Eight studies specified the use of a red diode laser [4,11,13-15,17,19,20]. The wavelength ranged from 400 to 890 nm, with a modal wavelength of 660 nm. In many of the control and treatment groups in the studies, additional wound treatment methods were utilised, including surgical debridement, saline irrigation and antibiotics.

Wound Size Reduction

All studies reported a reduction in the size of the wound in both the treatment and control groups. The wounds analysed were ulcers, burns and graft donor sites. Among the eight studies evaluating the percentage reduction in wound size, only Barreto and Salgado showed a greater reduction in the control group compared to the treatment group [24]. This was for ulcers in leprosy patients where both groups also received conventional treatments such as simple saline dressings with sterile gauze and 1% silver sulfadiazine cream, suggesting this may have been beneficial alone in the reduction of ulcer size. The remaining studies unanimously showed a higher rate of wound healing in the LLLT group with statistically significant results. In these studies, there was no correlation between wound size reduction and the wavelength or energy density of the laser. A previous meta-analysis by Woodruff et al. on laser therapy in wound repair on both human and non-human subjects found that energy density gave predictable dose-dependent treatment effects, and treatment effect size was greater in animals than in humans [8].

Diabetic Foot Ulcers

A third of the wounds analysed in the study were DFUs, mirroring the high prevalence within the United Kingdom patient demographic. In general, DFUs are poorly managed, and conventional treatment is often unsuccessful, highlighted by an amputation rate of around 7%-20% [3]. Standard treatment involves a multidisciplinary approach with glycaemic control, wound offloading, regular vascular assessment, infection control and wound debridement. Despite these measures, DFUs can be particularly recalcitrant and may require extended periods to heal [28]. LLLT not only enhances the healing of a wound but also promotes the healing of non-healing wounds and ulcers [8]. The meta-analysis by Huang et al. looked at LLLT on DFUs including 13 RCTs with 413 patients analysed and found that LLLT increases the rate of complete wound healing (p < 0.0001), reduces the ulcer area (p = 0.0002) and shortens the mean healing time (p < 0.0001) [3]. This is consistent with the sub-analysis of the six studies looking at LLLT on DFUs and one leprosy-induced ulcer, which also demonstrated a greater reduction in the size of the ulcers in the treatment group (p < 0.0001) [4,10,15,21,23,24]. A study by Salvi et al. investigated the vascular effects in DFUs after LLLT. They measured the total haemoglobin (Hb) concentration change before and after LLLT between a healthy control and intervention group. They found increased levels of total Hb after LLLT in DFU patients but not in the healthy controls, indicating enhanced perfusion to the wound, which promotes healing [29].

Episiotomy

The evidence suggests that LLLT is not beneficial for episiotomy wounds with limited change in the perineal pain scores. The RCT by Santos et al. [19] randomised participants into three groups, with two experimental groups and a control group. The experimental groups were split into treatments of LLLT with red and infrared lasers with wavelengths of 660 and 780 nm, respectively. The experimental group receiving the infrared laser showed a lower mean post-therapeutic pain score, suggesting that this is more effective at reducing pain than the red diode laser variant of LLLT [19]. The study by Alvarenga et al. was the only one of the three that showed a higher mean pain score in the treatment group than in the control post-LLLT, indicating that the treatment did not show any substantial benefit with scores that were both low and under 1.5 [16]. Treatment was delivered over one or three sessions postpartum, and there was no correlation in pain scores between the number of sessions given.

Limitations

There are limitations to this analysis, including the fact that a third of the skin wounds are DFUs. However, the results do highlight the potential for LLLT use within DFUs. This has potentially significant consequences in the management of these patient cohorts and can aid in reducing the morbidity and economic impact of managing complex diabetic wounds.

## Conclusions

Unlike other forms of medical laser therapy, LLLT is non-invasive and does not have thermal or ablative mechanisms. In this analysis, LLLT was demonstrated to have therapeutic effects on the body through the promotion of wound healing and pain alleviation. It has further illustrated its efficacy in treating chronic or slow-healing wounds such as DFUs. It would be beneficial to determine the parameters for the LLLT, including the wavelength, output power and energy density, which provide optimal treatment effects. The authors recommend conducting additional high-quality trials to further broaden the existing evidence base.
